# Supplementary material for: Specimen alignment with limited point-based homology: 3D morphometrics of disparate bivalve shells (Mollusca: Bivalvia)
Source: PeerJ. 2022 Jun 24;10:e13617. doi: 10.7717/peerj.13617 (PMC9235814; doi:10.7717/peerj.13617)
Supplement: Supplemental Information 1 [file peerj-10-13617-s001.pdf]

Supplementary Material

Specimen alignment with limited point-based homology: 3D morphometrics of disparate bivalve shells (Mollusca: Bivalvia)

Stewart M. Edie, Katie S. Collins, David Jablonski

S1. Supplemental Data

Table S1. Taxa used in this study and source of material. Museum acronyms: FMNH, Field Museum of Natural History; NHMUK, Natural History Museum, U.K.; USNM, U.S. Natural History Museum (also NMNH).

family	genus	species	authority	valve	museum	catalog no.	biv3d.meshid	morphosource_ark
Cardiidae	*Tridacna*	*squamosa*	(Lamarck 1819)	L	FMNH	166020	317	ark:/87602/m4/429843
Cuspidariidae	*Cuspidaria*	*rostrata*	(Spengler 1793)	L	USNM	811161	1272	ark:/87602/m4/429837
Glycymerididae	*Glycymeris*	*glycymeris*	(Linnaeus 1758)	L	USNM	199801	1683	ark:/87602/m4/429849
Mytilidae	*Modiolus*	*modiolus*	(Linnaeus 1758)	R	FMNH	126621	570	ark:/87602/m4/429855
Nuculanidae	*Nucula*	*pernula*	(Mueller 1779)	L	NHMUK	20180321	3255	ark:/87602/m4/429831
Ostreidae	*Ostrea*	*capsa*	J. G. F. Fischer von Waldheim 1807	R	FMNH	279417	138	ark:/87602/m4/429862
Pectinidae	*Pecten*	*maximus*	(Linnaeus 1767)	R	USNM	25529	1566	ark:/87602/m4/429868
Pholadidae	*Pholas*	*dactylus*	Linnaeus 1758	L	USNM	337277	2380	ark:/87602/m4/429874
Solecurtidae	*Tagelus*	*plebeius*	(Lightfoot 1786)	L	FMNH	177579	769	ark:/87602/m4/429894
Solenidae	*Ensis*	*siliqua*	(Linnaeus 1758)	L	USNM	27141	3144	ark:/87602/m4/429888
Veneridae	*Chione*	*elevata*	(Say 1822)	L	FMNH	176349	180	ark:/87602/m4/429881

S2. Supplemental Methods

S2.1. Placement of axis landmarks

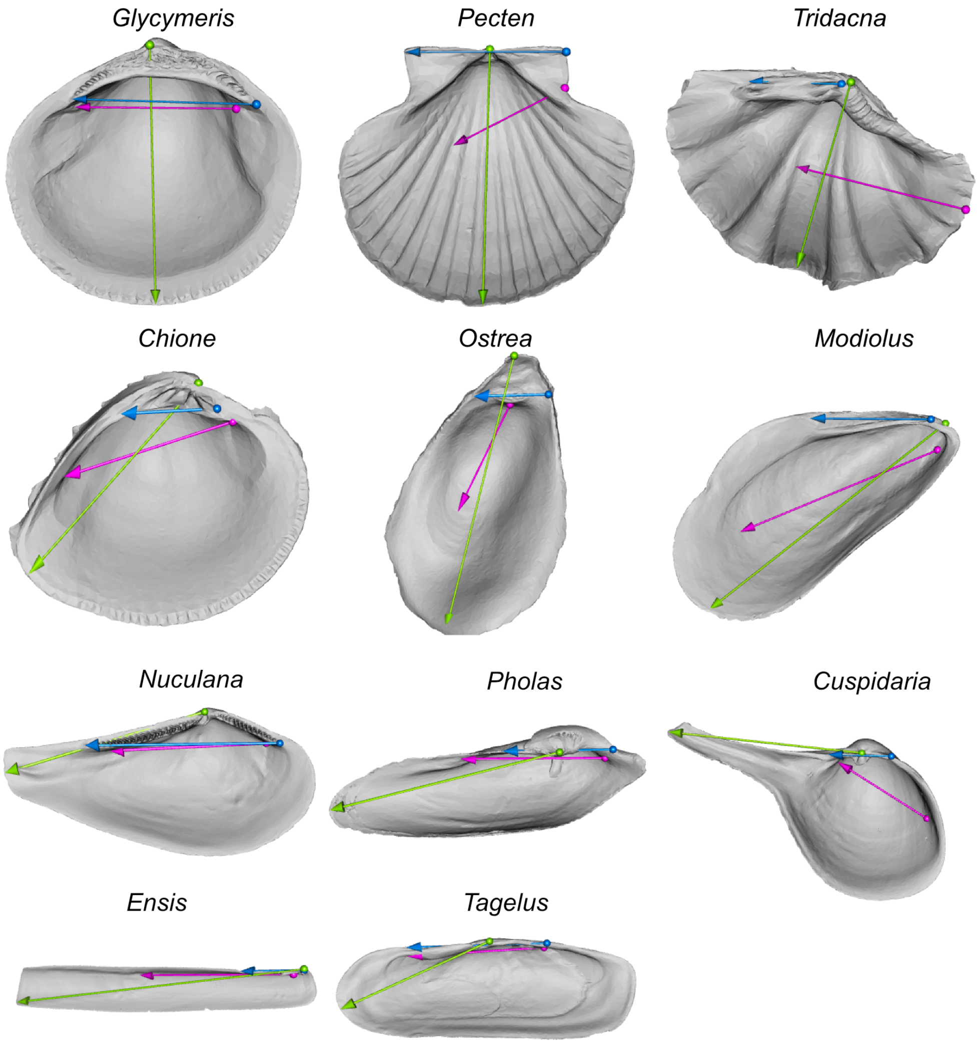

Figure S1. Placement of landmarks for axes (blue = hinge line; green = growth axis; magenta = oro-anal axis). Origins of axis vectors as spheres, termini as arrowheads. For the hinge line and oro-anal axis, spheres are anterior and arrowheads are posterior. For the growth axis, spheres mark the beak and arrowhead the farthest linear distance from the beak to a point on the commissure.

S2.2. Fitting the commissural plane

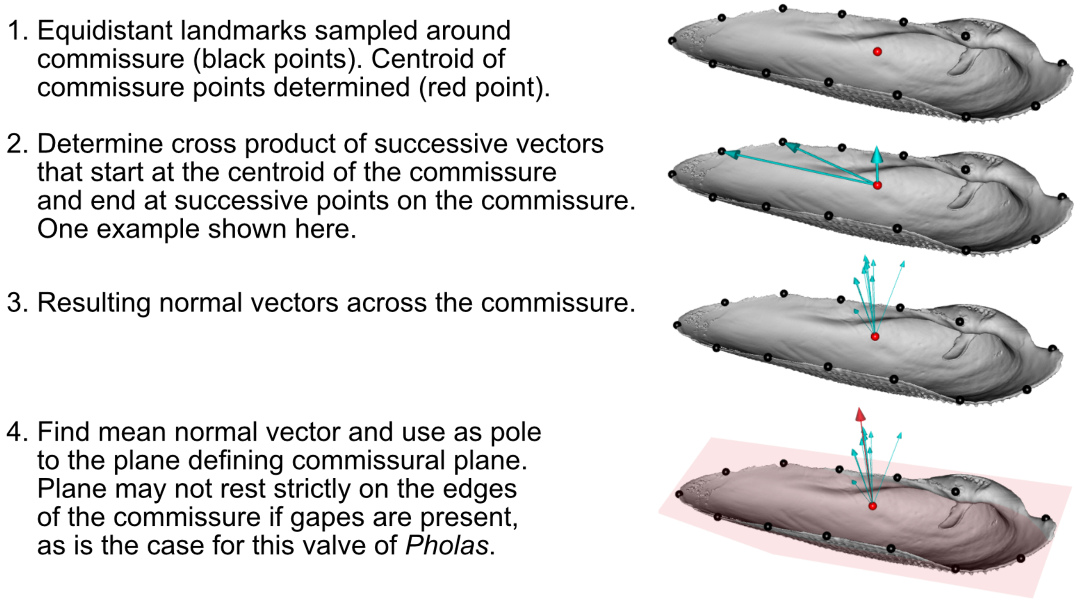

Figure S2. Visualization of the procedure for fitting the commissural plane.

S2.3. Landmarking the interior surface of the shell

First, the triangular surface mesh of the shell is 'cut' into two pieces using the commissure curve: (1) interior (facing the commissural plane, or proximally directed on the sagittal axis) and (2) exterior (facing away from the commissural plane, or distally directed on the sagittal axis); visualization and step-by-step details in Figure S3.

Then, equidistant surface semilandmarks are placed on the 'interior' surface of the shell mesh as described in Figure S4 (inspired by the eigensurface method of Polly and MacLeod 2008). Note that in Figure S4-Step 5, sorting points on a flat surface best handles the ordering of points on the often topographically complex and recurved surfaces, which, in our experience, confound sorting in three-dimensions. This process is imperfect, but, again, in our experience, more reliably captures the morphology of shell surfaces compared to atlas-based approaches (Schlager 2017; Bardua et al. 2019). Bardua et al. (2019:22) state: "more accurate placement of surface points is a far more biologically sound characterization of morphology than spurious placement"—which is why we used the gridded approach in Figure S4 to place the initial semilandmarks. After placement, the equidistant semilandmarks on each individual are slid to minimize their thin-plate spline (TPS) bending energy to the mean Procrustes shape (Gunz et al. 2005; Gunz and Mitteroecker 2013; implemented via *Morpho::slider3d* Schlager 2017). The start point of the commissure curve and the orientation of the surface semilandmark grid depend on the orientation scheme:

- For the commissure orientation (SX-COMM), the initial and 'fixed' (i.e. non-sliding) point of the 50-point, equidistant commissure curve is the point nearest the beak (Figure S5e). The other 49 semilandmarks along the commissure curve are then slid to minimize their TPS bending energy. The surface semilandmark grid is laid down at 5% distances along an arbitrary sampling axis that spans the 13th and 38th sliding semilandmarks on the commissure curve, which generally reflect the anterior and posterior directions, respectively. The outermost grid points that intersect the commissure of the valve are removed because they will be replaced by the sliding commissure semilandmarks in the final set. The semilandmark grid is then slid to minimize its TPS bending energy, using the sliding semilandmarks on the commissure curve to constrain the sliding of the surface semilandmarks. All sliding semilandmarks are constrained to lie on the mesh surface. Thus, the final landmark set consists of 50 sliding semilandmarks along the commissure and 380 sliding semilandmarks on the interior surface of the shell, totaling 430 sliding semilandmarks.
- For the oro-anal axis orientation (SX-OAX-oOAX), the initial and 'fixed' point of the 50-point, equidistant commissure curve is the point that forms the smallest angle between the orthogonal oro-anal axis vector and a vector originating at the centroid of the commissure curve and terminating at a point along it (Figure S5b). The aim is to reduce the impact of the beak position on the shape of the shell, that is, to remove the effects of shell growth on comparisons on its shapes. The sampling axis for the surface semilandmarks is the oro-anal axis. The commissure curve and surface semilandarks are slid as above.
- For the orientations that include the hinge line (SX-HL-oHL, SX-HL-GX, and SX-HL-GX-OAX), the initial and 'fixed' point of the 50-point, equidistant commissure curve is the point that forms the smallest angle between the orthogonal hinge line vector and a vector originating at the centroid of the commissure curve and terminating at a point along it (Figure S5a,c,d). The aim is the same as for the oro-anal axis above, and the semilandmarks are slid as in the SX-COMM case above.

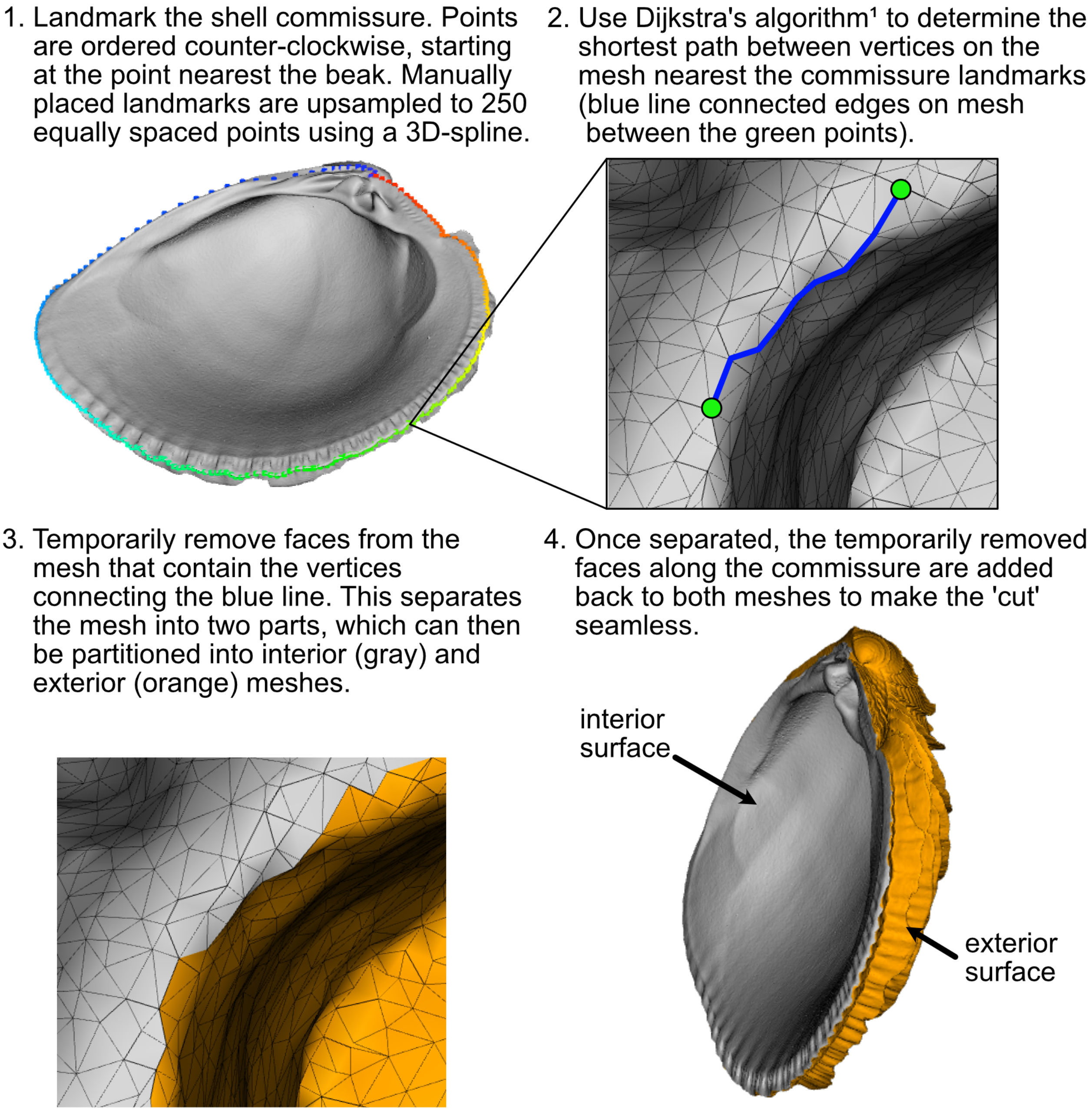

Figure S3. Visualization of the process for separating, or 'cutting,' shell meshes into interior and exterior surfaces. [1]Dijkstra, E.W. 1959. A note on two problems in connexion with graphs. Numerische Mathematik. 1:269–271.

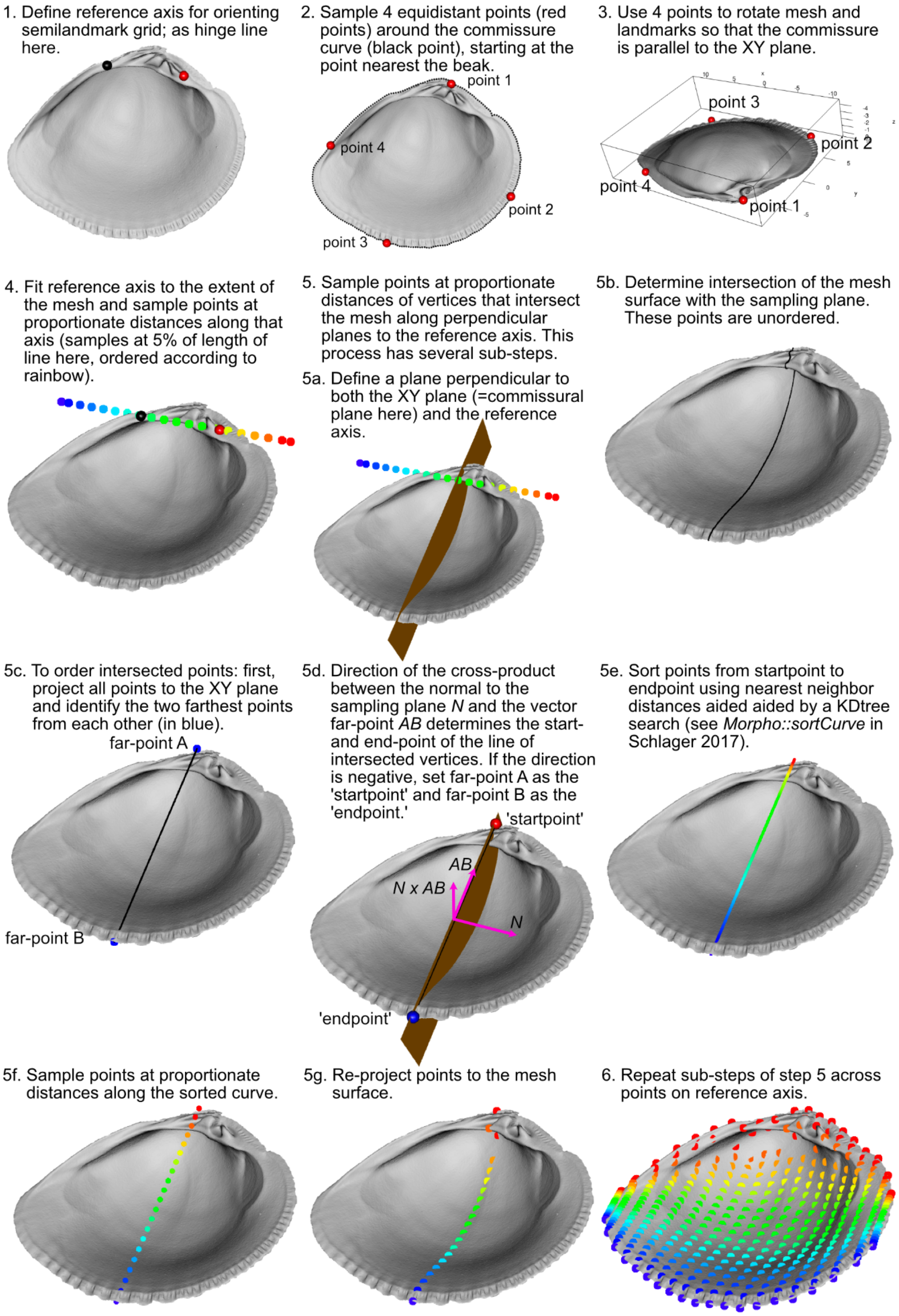

Figure S4. Visualization of the process for placing equidistant surface semilandmarks on the interior surface of the shell.

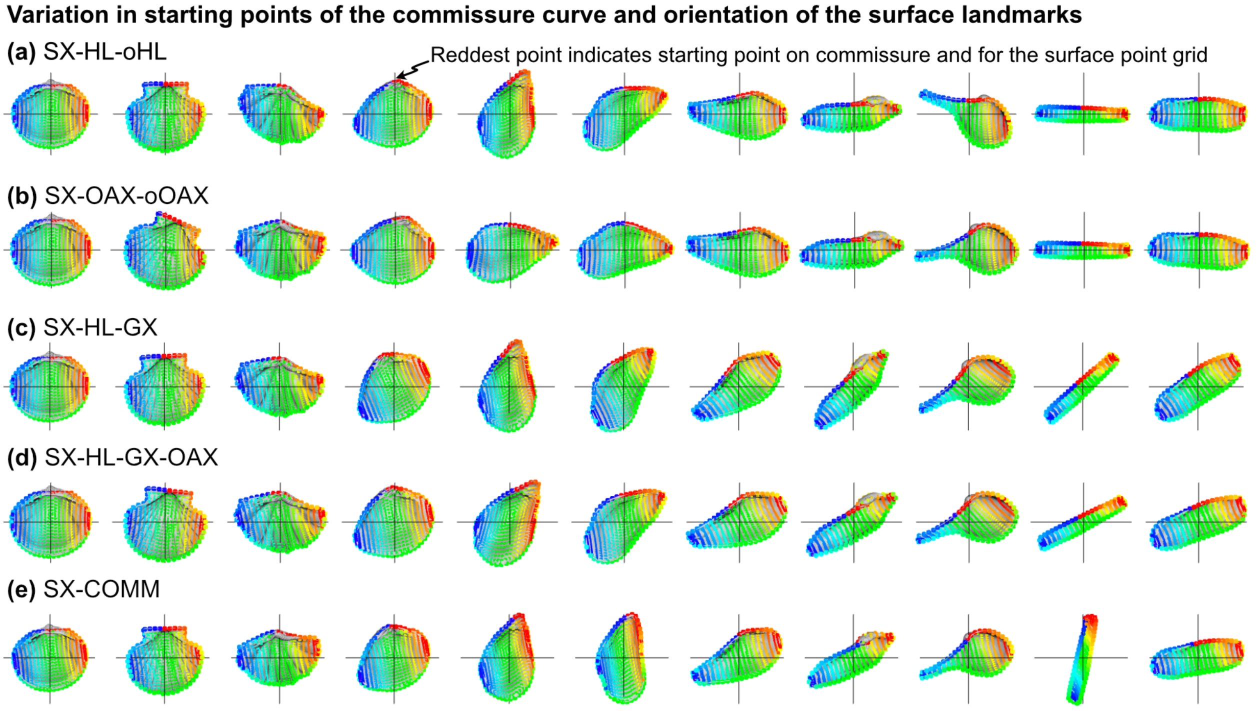

Figure S5. Placement of sliding semilandmarks along the commissure curve and the interior surface of the shell depending on orientation scheme. All landmark sets in this figure are scaled by the centroid size of the shell points and translated to the centroid of the shell commissure. Rainbow colored points indicate point order, with the most saturated red and blue as the respective initial and terminal points. (a) Commissure curve begins at the point that forms the smallest angle between the orthogonal hinge line vector and a vector originating at the centroid of the commissure curve and terminating at a point along it. Surface semilandmarks are oriented orthogonal to the hinge line. (b) Commissure curve begins at the point that forms the smallest angle between the orthogonal oro-anal axis vector and a vector originating at the centroid of the commissure curve and terminating at a point along it. Surface semilandmarks are oriented orthogonal to the oro-anal axis. (c) Commissure curve and surface landmarks oriented as in panel a. (d) Commissure curve and surface landmarks oriented as in panel a. (e) Commissure curve begins as the point nearest the beak. Surface semilandmarks are oriented orthogonal to the line connecting the 13th and 38th sliding semilandmarks on the commissure curve, which generally reflect the anterior and posterior directions.

S3. Supplemental Results

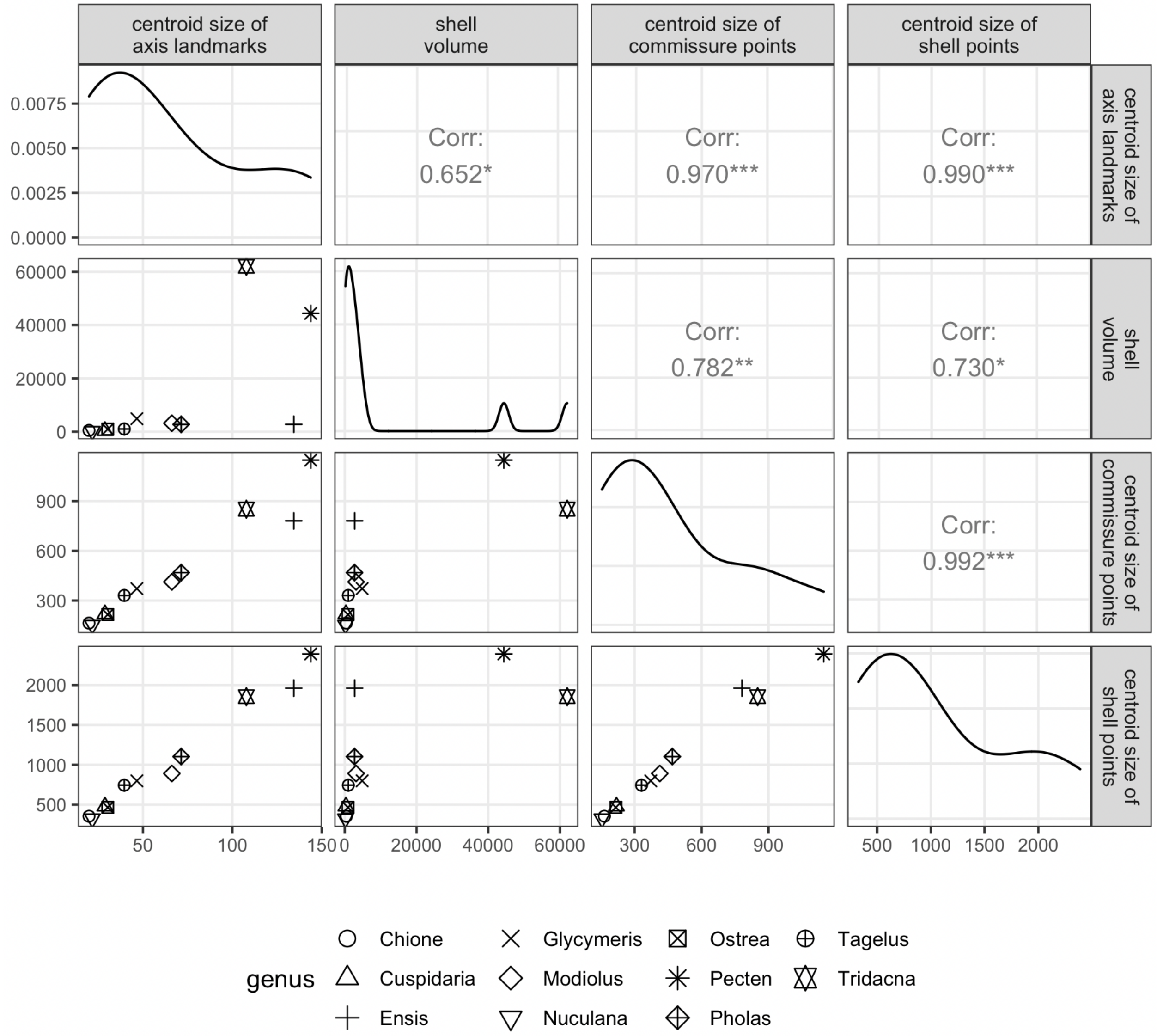

Figure S6. Correlations of size measures. Lower left triangle of the plot matrix shows the pairwise, bivariate relationships of size measures among analyzed specimens. Diagonal of the plot matrix shows density function for each size measure. Upper right triangle of the plot matrix shows results of Pearson correlation tests, with asterisks denoting significance at the following *p* levels: * = 0.05, ** = 0.01, and *** = 0.001.

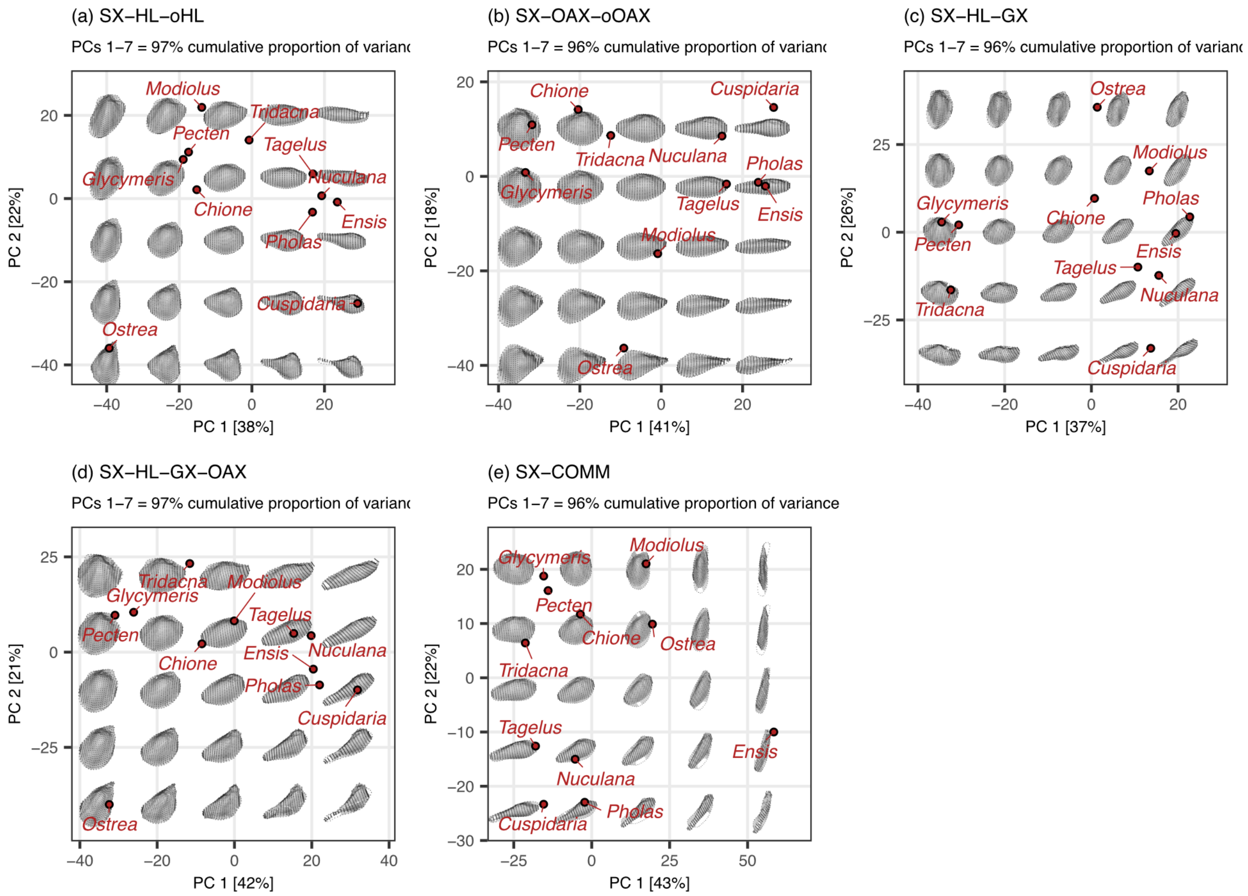

Figure S7. Principal components analysis of the aligned sliding semilandmarks on the commissure and interior surface of the shell. All landmark sets in this figure are scaled by the centroid size of the shell points and translated to the centroid of the shell commissure. Panels a-e give the positions of specimens on the first two principal components (PCs; percentages in brackets on each axis give the proportion of total variance explained by that axis). Images of shells are projections of the shapes at their given locations in the PC1-PC2 space. Holes in the mesh surfaces are artifacts of the meshing algorithm; the black points are the true underlying data.

S4. Supplemental References

Bardua, C., R. N. Felice, A. Watanabe, A.-C. Fabre, and A. Goswami. 2019: A practical guide to sliding and surface semilandmarks in morphometric analyses. Integrative Organismal Biology 1:obz016.

Gunz, P., and P. Mitteroecker. 2013: Semilandmarks: A method for quantifying curves and surfaces. Hystrix, the Italian Journal of Mammalogy 24:103–109.

Gunz, P., P. Mitteroecker, and F. L. Bookstein. 2005: Semilandmarks in three dimensions. Pp.73–98 *in* D. E. Slice, ed. Modern Morphometrics in Physical Anthropology. Kluwer

Academic Publishers-Plenum Publishers, New York.

Polly, P. D., and N. MacLeod. 2008: Locomotion in fossil carnivora: An application of eigensurface analysis for morphometric comparison of 3D surfaces. Palaeontologia Electronica 11:1–13.

Schlager, S. 2017: Morpho and Rvcg – Shape analysis in R: R-Packages for geometric morphometrics, shape analysis and surface manipulations. Pp.217–256 *in* G. Zheng, S. Li, and G. Székely, eds. Statistical Shape and Deformation Analysis: Methods, Implementation and Applications. Academic Press, London, UK.
